# An Ileal Crohn's Disease Gene Signature Based on Whole Human Genome Expression Profiles of Disease Unaffected Ileal Mucosal Biopsies

**DOI:** 10.1371/journal.pone.0037139

**Published:** 2012-05-14

**Authors:** Tianyi Zhang, Bowen Song, Wei Zhu, Xiao Xu, Qing Qing Gong, Christopher Morando, Themistocles Dassopoulos, Rodney D. Newberry, Steven R. Hunt, Ellen Li

**Affiliations:** 1 Department of Applied Mathematics and Statistics, Stony Brook University, Stony Brook, New York, United States of America; 2 Department of Medicine, Stony Brook University, Stony Brook, New York, United States of America; 3 Department of Medicine, Washington University-St. Louis School of Medicine, Saint Louis, Missouri, United States of America; 4 Department of Surgery, Washington University-St. Louis School of Medicine, Saint Louis, Missouri, United States of America; Institute for Genome Sciences - University of Maryland School of Medicine, United States of America

## Abstract

Previous genome-wide expression studies have highlighted distinct gene expression patterns in inflammatory bowel disease (IBD) compared to control samples, but the interpretation of these studies has been limited by sample heterogeneity with respect to disease phenotype, disease activity, and anatomic sites. To further improve molecular classification of inflammatory bowel disease phenotypes we focused on a single anatomic site, the disease unaffected proximal ileal margin of resected ileum, and three phenotypes that were unlikely to overlap: ileal Crohn's disease (ileal CD), ulcerative colitis (UC), and control patients without IBD. Whole human genome (Agilent) expression profiling was conducted on two independent sets of disease-unaffected ileal samples collected from the proximal margin of resected ileum. Set 1 (47 ileal CD, 27 UC, and 25 Control non-IBD patients) was used as the training set and Set 2 was subsequently collected as an independent test set (10 ileal CD, 10 UC, and 10 control non-IBD patients). We compared the 17 gene signatures selected by four different feature-selection methods to distinguish ileal CD phenotype with non-CD phenotype. The four methods yielded different but overlapping solutions that were highly discriminating. All four of these methods selected FOLH1 as a common feature. This gene is an established biomarker for prostate cancer, but has not previously been associated with Crohn's disease. Immunohistochemical staining confirmed increased expression of FOLH1 in the ileal epithelium. These results provide evidence for convergent molecular abnormalities in the macroscopically disease unaffected proximal margin of resected ileum from ileal CD subjects.

## Introduction

Transcriptomic analyses have highlighted differences in intestinal gene expression patterns between samples collected from patients with inflammatory bowel disease (IBD) compared to control patients without inflammatory bowel disease [1]–[11]. Differences in transcript levels, particularly those involved in inflammatory pathways, have been observed in macroscopically disease affected regions of the intestine compared to disease-unaffected regions of the intestine [6]. Molecular characterization of inflammatory bowel disease phenotypes based on transcriptomic analysis has been limited by sample heterogeneity with respect to disease phenotype, disease activity and anatomic sites. Most of the previous studies have focused on the colon, since this anatomic site is more easily accessible by colonoscopy.

We have previously examined genome wide expression profiles in the disease unaffected proximal margin of resected ileum collected from 4 patients with Crohn's disease of terminal ileum (ileal CD) undergoing initial ileocolic resection with that of 4 control non-IBD patients undergoing initial right hemicolectomy or total colectomy [8]. We have focused on the ileal CD phenotype and excluded subjects with Crohn's Colitis, sincethese two subphenotypes have distinct molecular characteristics [12]. Increased expression of candidate genes such as *MUC1*, *DUOX2* and *DMBT1* expression and decreased expression of *C4orf7* (follicular dendritic cell secreted peptide) was confirmed by reverse transcriptase polymerase chain reaction of 18 ileal CD and 9 control non-IBD samples. We found that these alterations in gene expression were independent of NOD2 genotype [8].

To better define the molecular characteristics of the ileal CD phenotype, we applied four different feature selection methods to select 17-gene signatures that would distinguish samples of the proximal disease unaffected proximal margin of ileum that were resected from individuals with ileal CD phenotype, from samples collected from non-CD phenotype (both non-IBD and ulcerative colitis patients) to a training set composed of 99 expression profiles. We then tested these features in an independently collected test set of 30 expression profiles.

## Materials and Methods

### Patients and Acquisition of Ileal Tissue Samples

This study was approved by the Washington University-St. Louis and Stony Brook University Institutional Review Boards. Ileal CD patients undergoing ileocolic resection, UC patients undergoing total colectomy and Control non-IBD patients undergoing either right hemicolectomy or total colectomy (for colon cancer, colonic adenomas, colonic inertia, diverticulosis and one case of a foreign body with perforation) were prospectively enrolled in a consecutive fashion by the Washington University Digestive Diseases Research Core Center Tissue Procurement Facility to donate surgically resected tissue samples between September 2005 and December 2010. A subset of 8 of the 99 expression profiles generated from samples collected between September 2005 and February 2010 in the training set were previously reported [8]. A subset of 81 of 99 expression profiles in the training set (Set 1) were previously reported in a study linking ileum associated microbial composition with cluster centroids corresponding to a cluster enriched in genes expressed in Paneth cells and two clusters enriched in genes associated with xenobiotic metabolism [11], [13]. The 30 expression profiles in the test set (Set 2) were collected from additional subjects recruited between February 2010 and December 2010. The diagnosis of CD or UC was based on the surgical pathological report for the surgical resection specimen, which was issued by the attending surgical pathologist assigned to the case. Patients who were unwilling or unable to give informed written consent were excluded. At least 4 ex-vivo biopsies were collected from the macroscopically disease-unaffected proximal margin of the freshly resected pathologic ileum specimens using Radial Jaw4 large-capacity biopsy forceps (Boston Scientific, Natick, MA) and placed immediately into RNAlater, an RNA stabilization solution, and stored at -80°C. The designation of *disease-unaffected* was based on the macroscopic appearance of the ileal mucosa and the surgical pathology report of adjacent ileal biopsies (“no histopathologic abnormality”). The clinical information and samples were collected as previously described [11], [13] and stripped of all identifying information and assigned both a patient code and sample code. All of the patients received intravenous antibiotic prophylaxis covering both aerobic and anerobic bacteria (e.g. ciprofloxacin and metronidazole, cefoxitin, cefotetan) within one hour of incision [14].

### Microarray Analysis

Total RNA was extracted from the tissue samples using TRI Reagent® according to the manufacturer's recommendation, and RNA quality was assessed using an Agilent 2100 Bioanalyzer [8]. The test RNAs and a common reference ileal RNA were labeled with the Quick Amp Labeling Kit (Agilent No. 5190-0424) and the resulting probes were hybridized to Agilent Whole Human Genome Arrays (Agilent No. G4412A) as previously described [8], [9]. The pre-processing, filtering and normalization of the microarray data was conducted using the R package LIMMA [15], [16] Probes with all Genepix flags less than −100 were treated as absent and removed from the dataset. There were technical duplicates on three samples in the training set and two samples in the test set. For those samples, the log2 ratios for the technical duplicates were averaged prior to analysis. The data discussed in this publication have been deposited in NCBI's Gene Expression Omnibus and are accessible through GEO Series accession number GSE24287 (http://www.ncbi.nlm.nih.gov/geo/query/acc.cgi?acc=GSE24287).

### Statistical Analysis

Two-class (ileal CD vs. non-CD) unpaired significance analysis of microarrays (SAM) was performed on 25,756 probes in the training set as previously described by Tusher *et al* [17] as an initial filtering step (>1.5 fold, <0.67 fold, FDR <0.05). SAM assigns a gene-specific t-test (q-value) based on changes in gene expression relative to the standard deviation of repeated measurements for that gene. Feature subset selection of 17-gene signatures was performed on the resulting 464 probes selected by SAM using the following four methods: Component-wise Boosting (Boosting) [18], Prediction Analysis of Microarrays (PAM) [19], Random Forest [20] and Least Absolute Shrinkage and Selection Operator (LASSO) [21]. In order to evaluate the four different feature selection methods, a majority vote [22] based on the median score of seven supervised machine learning tools, Boosting [18], PAM [19], Random Forest [20], LASSO [21], Support Vector Machine [23], Linear Discriminant Analysis [24], Naive Bayes [25]), was performed. The overall accuracy, sensitivity, specificity and area under the curves (AUC) were initially calculated based on the empirical receiver operating characteristic (ROC) curves [26]. The ROC curves were then smoothed to facilitate visual differentiation as previously described [27]. Partial correlation network analysis based on the joint sparse regression models [28] was further conducted to study the network relationship among the 17 gene signature selected by the boosting method.

### Immunohistochemistry

Folate hydrolase 1 (FOLH1), also termed prostate specific membrane antigen (PSMA) [29], expression in formalin fixed paraffin embedded sections of the disease unaffected proximal margin of resected ileum from ileal CD patients and Control non-IBD patients, were stained using a monoclonal mouse anti-PSMA antibody (clone E6, catalog number N1611, DAKO) in the Washington University Digestive Diseases Research Core Center Morphology Core. Epitope retrieval was performed with the Diva DECLOAKER reagent (BIOCARE DV-2004) in a Biocare Decloaking chamber. Primary antibody was applied overnight at 4°C at a dilution of 1∶500. Antigen antibody complexes were detected with biotinylated goat anti-mouse IgG (1∶2000, Jackson Laboratories), then developed in diaminobenzimidine (Biocare Betazid DAB) and counterstained with hematoxylin. Negative control slides were incubated with isotype-matched immunoglobulin, and a prostatic adenocarcinoma specimen served as a positive control for staining with the anti-PSMA (FOLH1) antibody.

## Results

### Patient Characteristics in the Training and Test Sets (see Table 1)

**Table 1:** Patient characteristics associated with each disease phenotype in the training and test sets.

Training Set			
Variables	Ileal CD (n = 47)	UC (n = 27)	Control (n = 25)
Gender (male)	43%	59%	32%
Race (white)	96%	100%	96%
Median Age (range) y	35 (20–75)	43 (17–64)	55 (18–84)
Current smoker	32%	10%	24%
Positive fecal *C. difficile* toxin	0%	30%	0%
Median BMI (range) kg/m^2^	24 (16–38)	24 (18–43)	28 (20–38)
5-ASA	55%	63%	0%
Steroids	43%	67%	0%
Immunomodulators	45%	44%	0%
Anti-TNFα biologics			
Current (≤8 weeks of surgery)	28%	41%	0%
Past (>8 weeks of surgery)	8%	7%	0%
Never	64%	52%	0%

Test Set			
Variables	Ileal CD (n = 10)	UC (n = 10)	Control (n = 10)
Gender (male)	40%	70%	40%
Race (white)	90%	90%	100%
Median Age (range) y	39 (19–58)	44 (16–62)	66 (21–77)
Current smoker	20%	20%	40%
Positive fecal *C. difficile* toxin	0%	30%	0%
Median BMI (range) kg/m^2^	22 (19–39)	24 (20–33)	25 (20–36)
5-ASA	40%	80%	0%
Steroids	70%	100%	0%
Immunomodulators	40%	30%	0%
Anti-TNFα biologics			
Current (≤8 weeks of surgery)	40%	20%	0%
Past (>8 weeks of surgery)	0%	0%	0%
Never	60%	80%	0%

The patients included in this study were predominantly white. As shown in Table 1, *C. difficile* was more prevalent among UC patients than ileal CD or control non-IBD patients [30], [31]. None of the control subjects were treated with5-ASA, immunomodulators, and/or anti-TNFα biologics. However all of the patients received intravenous antibiotic prophylaxis that covered both aerobic and anaerobic bacteria within one hour prior to incision [14].

### Two-Class Unpaired Significance Analysis of Microarrays (SAM) Comparing Ileal CD and Non-CD (UC and control non-IBD) Phenotypes (see Table S1)

Because a large amount of variability can be introduced in the fold change for low intensity signals, the threshold for gene filtering was selected to be twice the background, resulting in a total of 25,676 gene-probes [32]. Two-class unpaired SAM analysis comparing ileal CD with non-CD phenotype was performed as the initial filtering step, and identified 464 gene probes (see Table S1) that were differentially expressed (fold change ≥1.5 or ≤0.67, FDR <0.05) between ileal CD and Non-CD (UC and Control) samples [17]. In this training set of 99 microarrays, the mean *DMBT1* expression level was confirmed to be significantly increased, while that of *C4orf7* was confirmed to be significantly decreased in the disease unaffected proximal margin of ileum resected from ileal CD patients compared to nonIBD Control and UC patients [8]. We also observed that *MUC1* and *DUOX2* expression was increased relative to Control samples. However because *MUC1* and *DUOX2* expression was also increased in UC compared to nonIBD Control samples, these genes were not selected in this two-class unpaired SAM comparing ileal CD and non-CD (UC and Control).

### Feature Subset Selection (see Table S2)

Four feature subset selection methods (Boosting [18], PAM [19], Random Forest (RF) [20], and LASSO [21]), were applied to further select subsets of 17 gene probes or features that were useful for predicting the ileal CD phenotype. The union of the resulting four 17-gene signatures totaled 42 in number (see Table S2) because 26 of the features were selected by more than one method.Folate hydrolase 1 (*FOLH1*) gene was selected by all four feature selection methods. Three known genes, TLR4 interactor with leucine rich repeats (*TRIL*), Niemann-Pick disease, type C1, gene-like 1 (*NPC1L1*), and *C4orf7* also termed follicular dendritic cell secreted protein were selected by three of the four methods. Six known genes were selected by two of four methods, BCL2-associated X protein (*BAX*), cytochrome P 450, family 26, subfamily B, polypeptide 1 (*CYP26B1*), nephronectin (*NPNT*), protein phosphatase 1, regulatory (inhibitor) subunit 14A (*PPP1R14A*), family with sequence similarity129, member C (*FAM129C*) also termed B-cell novel protein 1 (*BCNP1*), cathelicidin antimicrobial peptide (*CAMP*), chemokine (C-C motif) ligand 23 (*CCL23*). We repeated our analysis using data excluding the *C. difficile* positive samples. *FOLH1* is still the only gene probe selected by all four feature selection methods and it is still ranked prominently by all four classifiers 2^nd^, 1,st, 1^st^ and 4^th^ by PAM, RF, LASSO and Boosting, respectively). In addition, ten out of 12 genes selected by two or more feature methods based on data without *C. difficile* positive samples overlap with those selected using data including the *C. difficile* positive samples. Meanwhile, the Bossting method still features the highest classification accuracy at 89.90% and 86.96% for data with and without the *C. difficile* positive samples, respectively. All these observations indicate that our method was not skewed by the *C. difficile* toxin factor.

**Figure 1:**
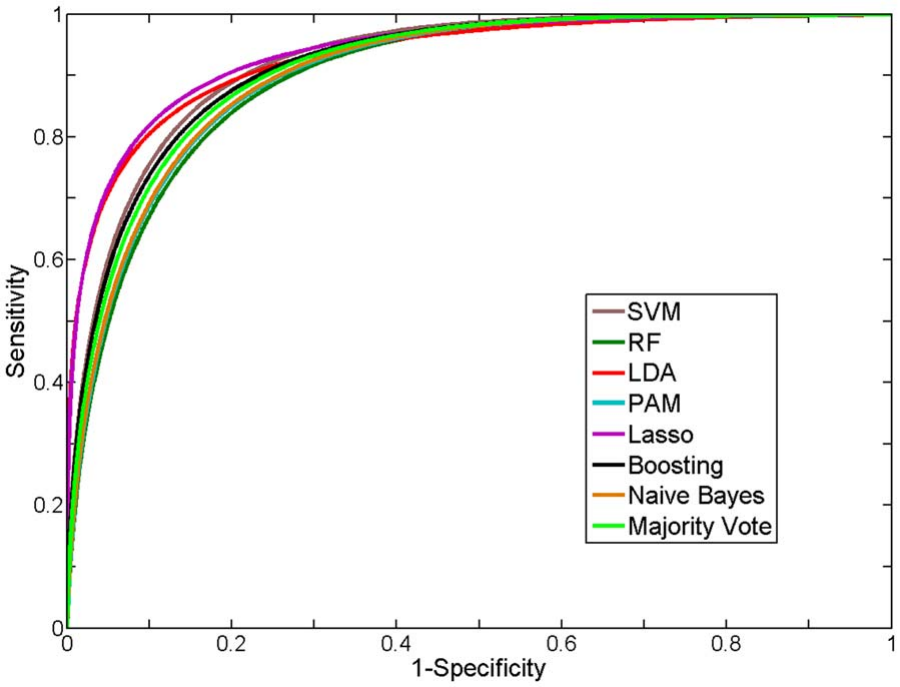
Receiver operating characteristic (ROC) curve for different classification methods on the training set.

**Figure 2:**
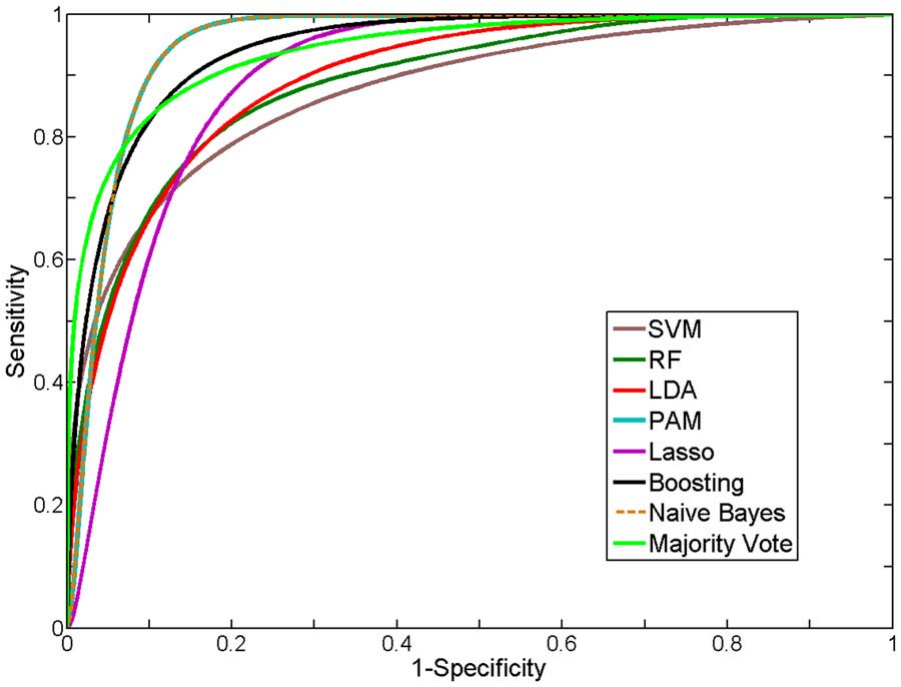
Receiver operating characteristic (ROC) curve for different classification methods on the test set.

Majority vote based on the median score of seven classifier tools (see Materials and Methods) was used to assess the accuracy associated with each feature subset for ileal CD phenotype in the training set via Jack-Knife (take-one-out) cross validation. The feature subset selected by the boosting method yielded the highest area under the curve (AUC) and overall accuracy (see Table 2). The smoothed receiver operating characteristic (ROC) curves for the seven classifiers as well as their majority vote based on the training data were comparable (see Figure 1). We then applied this 17 gene signature to an independent test set that was collected after the training set (see Figure 2, Table 3). As shown in Table S2, the polarity of the mean fold change for this 17 ileal gene signature was preserved in both the training and test set. Of note, errors in classification reflected misclassification of UC samples as ileal CD samples. The smoothed ROC curves are shown in Figure 2 in order to facilitate visual differentiation of the different classifiers. There was good agreement between the AUC for the empirical and smoothed ROC curves (see Table S3), indicating that the smoothed ROCs retained the key properties of the empirical ROCs.

**Table 2:** Comparison of 17 ileal gene signatures selected by four different feature selection methods.

Methods	AUC	Accuracy
**Boosting**	**0.928**	**89.9%**
PAM	0.895	88.9%
Random forest	0.902	85.9%
LASSO	0.895	85.9%

Boosting [16], PAM) [17], random forest [18] and LASSO [19] were applied to the SAM filtered training microarray dataset to select 17 ileal gene signatures. The AUC and overall accuracy for each of the signatures were calculated based on the majority vote of 7 classifiers (Boosting, PAM, Random Forest, LASSO, Support Vector Machine, Linear Discriminant Analysis, and Naive Bayes), which is equivalently to the decision based on the median score using an usual probability threshold of 0.5 (see Materials and Methods).

**Table 3:** Classification results on the training and test sets.

Classification Method	Accuracy	Sensitivity	Specificity
**Training Set**			
Support Vector Machine (SVM)	90.9%	91.5%	90.4%
Random Forest (RF)	86.9%	87.2%	86.5%
Linear Discriminant Analysis (LDA)	90.9%	89.4%	92.3%
Predictive Analysis of Microarray (PAM)	88.9%	89.4%	88.5%
Lasso	91.9%	91.5%	92.3%
Boosting	88.9%	89.4%	88.5%
Naïve Bayes	88.9%	89.4%	88.5%
***Majority Vote (Combined Classifiers)***	***89.9%***	***91.5%***	***88.5%***
**Test Set**			
Support Vector Machine (SVM)	83.3%	80.0%	85.0%
Random Forest (RF)	73.3%	90.0%	65.0%
Linear Discriminant Analysis (LDA)	76.7%	80.0%	75.0%
Predictive Analysis of Microarray (PAM)	86.7%	100.0%	80.0%
Lasso	86.7%	80.0%	90.0%
Boosting	86.7%	90.0%	85.0%
Naïve Bayes	83.3%	100.0%	75.0%
***Majority Vote (Combined Classifiers)***	***80.0%***	***90.0%***	***75.0%***

The accuracy, sensitivity, specificity of the ileal gene signature selected by the boosting method [16] are calculated using Leaving-One-Out cross validation on the training and subsequently, direct classification of the test set based on the training set.

### *FOLH1* is a “Hub” Gene by Partial Correlation Network Analysis

**Figure 3:**
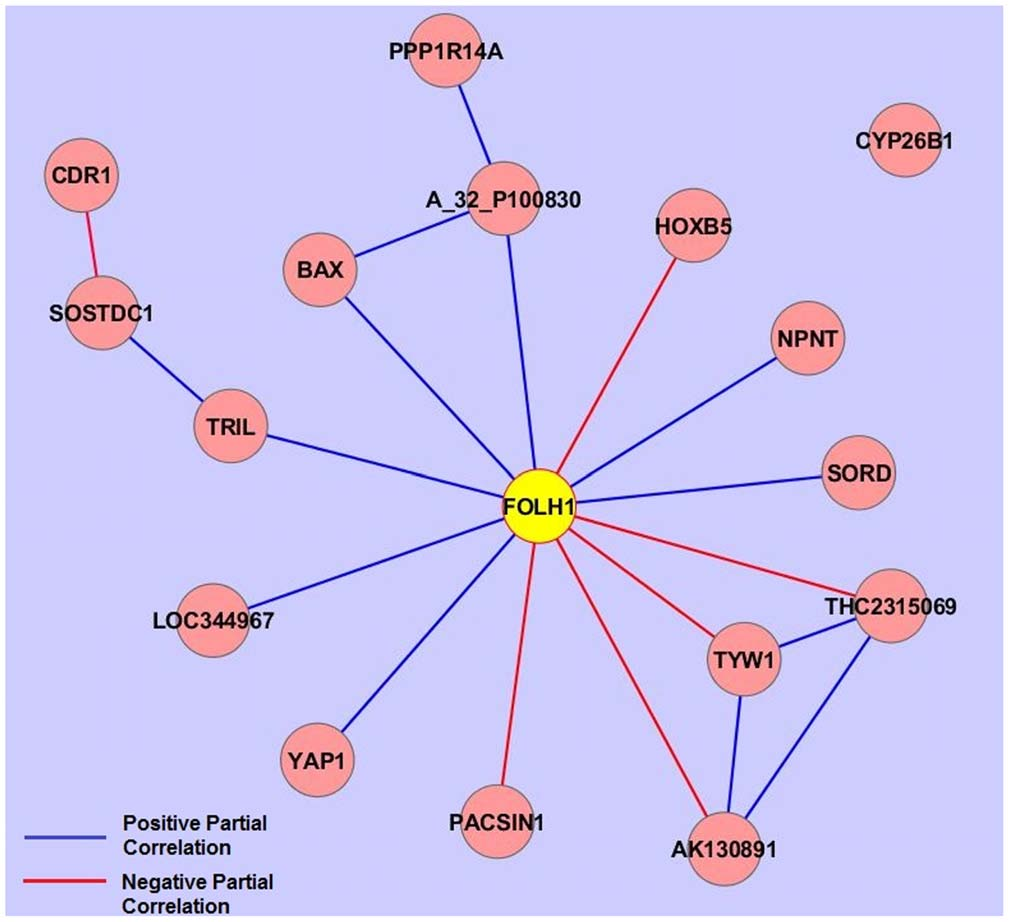
Partial correlation network among the 17 selected genes. FOLH1 is linked to multiple genes and serves as a hub gene. A red line between genes indicates a positive non-zero partial correlation and a blue line indicates a negative non-zero partial correlation.

**Figure 4:**
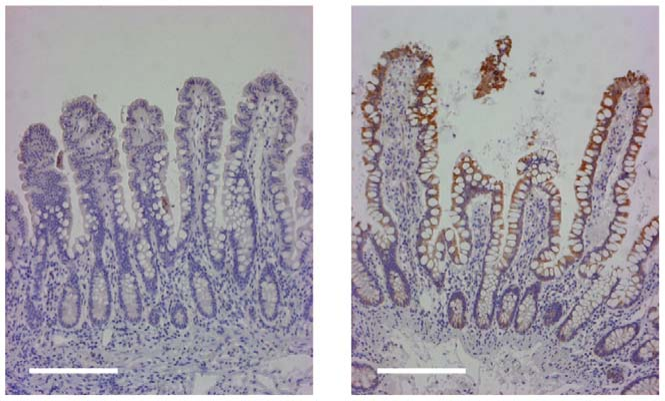
Immunohistochemical localization of FOLH1 in disease unaffected ileal mucosa from the proximal margin of resected ileum from an ileal CD subject (left panel) and a control non-IBD subject. The more prominent FOLH1 staining in the ileal CD sample is localized to the villous epithelium. Magnification is 100×. Bar is 200 µm.

Partial correlation network analysis was conducted on the union of the features selected by the four methods using all 129 microarrays in both the training and test set to assess the coregulation of these 42 genes. As shown in Figure 3, the folate hydrolase 1 (*FOLH1*) gene was identified as a “hub” gene that has significantly non-zero partial correlations to 12 of the other 16 gene biomarkers (see Figure 3). The *FOLH1* gene was originally identified as a prostate specific membrane antigen detected as upregulated in prostate carcinoma [33], however expression of *FOLH1* has since been observed in other tissues including the small intestine, particularly in the duodenal mucosa, the nervous system and the kidney [34]. Because *FOLH1* expression has been observed in neoplastic and nonneoplastic neovasculature [35], immunochemical localization of *FOLH1* was performed on the disease unaffected proximal margin of resected ileum from ileal CD and control non-IBD subjects. A representative micrograph is shown in Figure 4, which demonstrates that the more prominent staining in ileal CD samples was localized to the villous epithelium.

## Discussion

In this study, we took a statistical approach to identify ileal gene biomarkers associated with ileal CD phenotype compared to non-CD (UC and control). Some of the genes (e.g. *DUOX2* and *MUC1*) that we noted previously to be upregulated in ileal CD with control non-IBD subjects were not selected in the current study because these genes were also upregulated in UC compared to control samples [8]. Feature selection is one of the most important issues in classification. In this study, four feature selection methods, (Boosting, PAM, Random Forest and LASSO), were applied to select subsets of 17 gene features. The four methods yielded different but overlapping solutions that were highly discriminating. Thus, feature selection with microarray data can lead to different solutions that are comparable with respect to prediction rates. Note that different underlying hypotheses are associated with each method in selecting features from an extremely large number of variables in the microarray datasets compared to the number of samples [36], [37]. Combining different methods has been used as an approach to improve classification performance [38], [39].

All four selection methods identified upregulation of *FOLH1* expression as predictive of the ileal CD phenotype compared to non-CD. *FOLH1* encodes a transmembrane glycoprotein that acts as a glutamate carboxypeptidase on substrates including folate. Immunohistochemical staining localized more prominent expression of this gene in ileal CD samples to the villous epithelium [34]. Of the features selected by alternative feature selection methods (see Table 2), only *FOLH1B* clustered with *FOLH1* in the training dataset [11]. *FOLH1* is an established biomarker for prostate cancer, but has not been previously identified as a biomarker for Crohn's disease.

Three genes, *TRIL*, *NPCL1* and *C4orf7* were selected by three of four of the feature selection methods. *TRIL*, was recently identified as a novel component of the TLR4 complex and TLR3 complex [40], [41]. *TRIL* mRNA expression has been detected in the small intestine, as well as the central nervous system, lung, kidney and ovary. TRIL expression is upregulated in cell culture by lipopolysaccharide. The upregulation of *TRIL* expression could reflect altered host microbial interactions in macroscopically disease unaffected regions of the intestine in ileal CD patients. *NPC1L1* is required for intestinal uptake of cholesterol and plant sterols and is relatively abundant in the ileum [42], [43]. Upregulation of *NPC1L1* expression in ileal CD patients may also contribute to enhanced atherogenesis in Crohn's patients [44]. Partial correlation network analysis revealed that *FOLH1* has nonzero correlations with 12 of the other 16 genes in the signature. The biological basis for the nonzero partial correlations between the “hub” gene, *FOLH* is not immediately apparent.

We have previously noted downregulation of *C4orf7* as well as other genes associated with organized lymphoid structures and/or B-cell function in ileal CD patients compared to non-IBD control patients [8]. This study indicates that downregulation of ileal *C4orf7* expression is also observed in ileal CD patients, when compared with UC patients.

Partial correlation network analysis revealed that *FOLH1* has nonzero correlations with 12 of the other 16 genes in the signature. The biological basis for the nonzero partial correlations between the “hub” gene, *FOLH* is not immediately apparent. Thus far, we have not detected association of the gene features listed above with alterations in microbial composition, but we are likely underpowered to detect such associations with only 81 samples with paired microbiome and microarray data [11]. We also noted that upregulation of *FOLH1* was observed in ileal CD samples regardless of NOD2 genotype [8].In this study we report the results of binary classification – ileal CD vs. non-CD. Our attempts to apply multiclassification to the data set yielded poor accuracy particularly between the UC and control non-IBD phenotypes. This may be partly because the number of UC samples and control non-IBD samples were both smaller than the number of ileal CD samples. Of note, the errors in the binary classification of ileal CD vs. non-CD reflected misclassification of two UC samples as ileal CD. In the original test set we had an additional sample from a subject with a pre-operative diagnosis of UC. However the post-operative diagnosis was changed to Crohn's colitis based on the pathological diagnosis of the resected specimen. Interestingly this discarded sample was classified as “ileal CD” based on the expression profile. While the ileal CD phenotype can be easily distinguished from ulcerative colitis based on imaging and endoscopic findings, it is more difficult to distinguish Crohn's colitis from ulcerative colitis even after pathological diagnosis of the resected colon [45]. Improving our ability to distinguish UC from Crohn's colitis at the time of the initial colon resection would improve clinical decision making with respect to performing a subsequent ileal pouch anal anastomosis [46]. For this reason we are continuing to follow our UC patients after colectomy to determine whether any of these patients are diagnosed subsequently as Crohn's disease. We also plan to begin analyzing disease unaffected ileal samples collected from patients undergoing colectomy for Crohn's colitis to determine whether there is any overlap in the ileal signature for ileal CD and Crohn's colitis.

In summary, we have identified potential biomarkers for ileal CD phenotype in the macroscopically disease unaffected proximal margin of resected ileum from ileal CD subjects. These results provide evidence for convergent molecular abnormalities in the macroscopically disease unaffected proximal margin of resected ileum from ileal CD subjects,

## Supporting Information

Table S1 Differentially expressed gene probes selected by SAM. A. Upregulated probes in 47 ileal CD compared to 52 non-CD (UC and control non-IBD) samples (number of probes = 269). B. Downregulated probes in 47 ileal CD compared to 52 non-CD samples (total number of probes = 195).
(DOCX)

Table S2 Union of differentially expressed Agilent gene probes selected by four feature subset selection methods: Boosting, PAM, Random Forest (RF) and LASSO. A. Upregulated probes B. Downregulated probes The 17 genes selected by the boosting method are **bolded**.
(DOCX)

Table S3 AUC values of empirical ROC curves, AUC values and 95% confidence interval (C.I.) of smoothed ROC curves.
(DOCX)
