# Supplementary material for: An Ileal Crohn's Disease Gene Signature Based on Whole Human Genome Expression Profiles of Disease Unaffected Ileal Mucosal Biopsies
Source: PLoS One. 2012 May 14;7(5):e37139. doi: 10.1371/journal.pone.0037139 (PMC3351422; doi:10.1371/journal.pone.0037139)
Supplement: Table S1 — Differentially expressed gene probes selected by SAM. A. Upregulated probes in 47 ileal CD compared to 52 non-CD (UC and control non-IBD) samples (number of probes = 269). B. Downregulated probes in 47 ileal CD compared to 52 non-CD samples (total number of probes = 195). (DOCX) [file pone.0037139.s001.docx]

**Supplementary Table S1A. Gene probes upregulated in 47 ileal CD compared to 52 non-ileal CD (UC and control non-IBD) samples** (total number of probes = 269).

| **Agilent ID** | **Gene Name, Accession Number, Description** | **Fold-change** |
| --- | --- | --- |
| A_23_P47616 | FOLH1 (PSMA) | 6.26 |
| A_32_P157391 | PSMAL | 5.35 |
| A_23_P79217 | LCT | 3.69 |
| A_23_P20075 | NPC1L1 | 3.11 |
| A_23_P55828 | CCL25 | 2.3 |
| A_23_P106194 | FOS | 2.28 |
| A_23_P119763 | ABCG5 | 2.27 |
| A_23_P86599 | DMBT1 | 2.16 |
| A_24_P42264 | LYZ | 2.09 |
| A_23_P19663 | CTGF | 2.09 |
| A_23_P80491 | RBP2 | 2.08 |
| A_24_P53778 | ITLN2 | 2.06 |
| A_23_P255345 | VNN1 | 2.06 |
| A_23_P502464 | NOS2A | 2.05 |
| A_23_P2789 | OLFM4 | 2.05 |
| A_23_P114008 | TM4SF20 | 2.05 |
| A_24_P337700 | VNN1 | 2.04 |
| A_23_P46426 | CYR61 | 1.99 |
| A_24_P92683 | IGHA1 | 1.97 |
| A_23_P429998 | FOSB | 1.92 |
| A_23_P102864 | PRSS7 | 1.9 |
| A_24_P110242 | ENST00000360102 | 1.9 |
| A_24_P326084 | HLA-DQA1 | 1.9 |
| A_24_P341126 | A_24_P341126 | 1.9 |
| A_24_P702749 | Ig heavy chain variable region, VH3 | 1.9 |
| A_24_P144346 | Ig heavy chain variable region, VH3-7 | 1.89 |
| A_24_P229447 | ENST00000360102 | 1.89 |
| A_23_P46429 | CYR61 | 1.88 |
| A_24_P315941 | Ig M, VH3-N-D-N-JH4 | 1.88 |
| A_24_P384604 | A_24_P384604 | 1.88 |
| A_23_P149517 | PIGR | 1.86 |
| A_24_P852001 | ENST00000383048 | 1.86 |
| A_32_P65628 | REG3G | 1.86 |
| A_23_P19523 | MLN | 1.85 |
| A_23_P214080 | EGR1 | 1.85 |
| A_24_P323298 | LOC652159 | 1.85 |
| A_24_P384119 | LOC652791 | 1.85 |
| A_24_P161853 | LOC440361 | 1.84 |
| A_24_P100684 | ENST00000379895 | 1.82 |
| A_23_P19529 | MLN | 1.81 |
| A_23_P200728 | FCGR3A | 1.81 |
| A_23_P21249 | Ig heavy chain VHDJ region | 1.81 |
| A_24_P889462 | IGHA1 | 1.81 |
| A_23_P155786 | SULT1E1 | 1.8 |
| A_24_P101226 | ENST00000354689 | 1.8 |
| A_24_P15550 | Myosin-reactive Ig light chain variable region | 1.8 |
| A_24_P590547 | IGHA1 | 1.8 |
| A_32_P234459 | HLA-H | 1.8 |
| A_24_P15388 | Interferon, alpha-inducible protein 6 | 1.79 |
| A_24_P33341 | ENST00000360102 | 1.79 |
| A_24_P370946 | CYR61 | 1.79 |
| A_24_P925505 | CD36 | 1.79 |
| A_32_P722809 | IGKV1-5 | 1.79 |
| A_23_P156890 | TCF21 | 1.78 |
| A_23_P34915 | ATF3 | 1.78 |
| A_23_P7212 | CFI | 1.78 |
| A_23_P321949 | PLA2G2A | 1.77 |
| A_24_P100228 | XBP1 | 1.77 |
| A_24_P101771 | A_24_P101771 | 1.77 |
| A_24_P518369 | A_24_P518369 | 1.77 |
| A_23_P155755 | CXCL6 | 1.76 |
| A_23_P395438 | HTRA3 | 1.76 |
| A_23_P73328 | Ig A heavy chain variable region | 1.76 |
| A_24_P24053 | ENST00000360623 | 1.75 |
| A_24_P488083 | Ig M heavy chain variable region | 1.75 |
| A_23_P205959 | ALDH1A3 | 1.74 |
| A_23_P70539 | HLA-C | 1.74 |
| A_24_P376483 | HLA-A | 1.74 |
| A_32_P74409 | ENST00000339446 | 1.74 |
| A_23_P111583 | CD36 | 1.73 |
| A_23_P15727 | FKBP10 | 1.73 |
| A_23_P30848 | HLA-E | 1.73 |
| A_23_P47102 | ACY3 | 1.73 |
| A_23_P62890 | GBP1 | 1.73 |
| A_24_P263767 | ENST00000376793 | 1.73 |
| A_24_P263786 | BC022362 | 1.73 |
| A_24_P417352 | IGHM | 1.73 |
| A_23_P119936 | REG3A | 1.72 |
| A_23_P136026 | IGHA1 | 1.72 |
| A_23_P253896 | NPNT | 1.72 |
| A_23_P425681 | CCK | 1.72 |
| A_24_P414999 | LAPTM4B | 1.72 |
| A_32_P51988 | ENST00000379879 | 1.72 |
| A_23_P134347 | CPVL | 1.71 |
| A_23_P64721 | GPR109B | 1.71 |
| A_23_P82886 | DEFA6 | 1.71 |
| A_24_P212024 | ENST00000259219 | 1.71 |
| A_24_P298805 | ENST00000360102 | 1.71 |
| A_24_P76868 | Ig lambda variable region | 1.71 |
| A_23_P158481 | CYP2C19 | 1.7 |
| A_23_P208706 | BAX | 1.7 |
| A_23_P395001 | SLC2A12 | 1.7 |
| A_23_P7144 | CXCL1 | 1.7 |
| A_23_P12767 | CYP2C9 | 1.69 |
| A_23_P214408 | UNC93A | 1.69 |
| A_23_P37702 | TPSB2 | 1.69 |
| A_23_P44053 | A_23_P44053 | 1.69 |
| A_24_P626951 | ENST00000295410 | 1.69 |
| A_32_P39440 | Ig kappa locus | 1.69 |
| A_23_P121533 | SPON2 | 1.68 |
| A_23_P162739 | TSC22D1 | 1.68 |
| A_23_P383009 | IGFBP5 | 1.68 |
| A_23_P96191 | IGKV4 | 1.68 |
| A_24_P144314 | A_24_P144314 | 1.68 |
| A_24_P161933 | A_24_P161933 | 1.68 |
| A_24_P273679 | YAP1 | 1.68 |
| A_24_P508946 | LOC648674 | 1.68 |
| A_23_P114423 | RGN | 1.67 |
| A_23_P259763 | Ig heavy chain variable region, VH3 | 1.67 |
| A_23_P209251 | THC2316768 | 1.66 |
| A_23_P251453 | HNF4G | 1.66 |
| A_24_P253003 | WNT11 | 1.66 |
| A_24_P92472 | CFI | 1.66 |
| A_23_P126248 | RNF186 | 1.65 |
| A_23_P36445 | TMED2 | 1.65 |
| A_24_P156922 | SCP2 | 1.65 |
| A_24_P330263 | EDNRB | 1.65 |
| A_24_P58673 | REG4 | 1.65 |
| A_24_P604784 | Ig heavy chain variable region | 1.65 |
| A_32_P157927 | ENST00000283657 | 1.65 |
| A_23_P156425 | MAN1A1 | 1.64 |
| A_23_P29257 | H1F0 | 1.64 |
| A_23_P435390 | A_23_P435390 | 1.64 |
| A_23_P64938 | MBOAT5 | 1.64 |
| A_24_P204727 | Ig heavy chain variable region | 1.64 |
| A_24_P224684 | SULT2A1 | 1.64 |
| A_32_P65022 | ENST00000327926 | 1.64 |
| A_32_P94444 | PRSS2 | 1.64 |
| A_23_P160800 | NR0B2 | 1.63 |
| A_23_P167168 | IGJ | 1.63 |
| A_23_P19936 | KDELR2 | 1.63 |
| A_23_P7727 | HAPLN1 | 1.63 |
| A_24_P494425 | ENST00000377226 | 1.63 |
| A_24_P608268 | Ig G heavy chain V region, VH3-JH4b | 1.63 |
| A_24_P698136 | Member of the acyl CoA thioesterase family | 1.63 |
| A_32_P127153 | SORD | 1.63 |
| A_23_P111766 | A_23_P111766 | 1.62 |
| A_23_P207456 | CCL8 | 1.62 |
| A_23_P39590 | XDH | 1.62 |
| A_23_P428298 | UNC5CL | 1.62 |
| A_23_P5300 | CPS1 | 1.62 |
| A_23_P66635 | CCL11 | 1.62 |
| A_24_P110012 | HLA-L MHC, class I, L, pseudogene | 1.62 |
| A_24_P363711 | DEFA6 | 1.62 |
| A_23_P158484 | CYP2C19 | 1.61 |
| A_24_P101642 | Ig heavy chain V-III region VH26-like | 1.61 |
| A_24_P314159 | APP | 1.61 |
| A_24_P714134 | Similar to heterogeneous nuclear ribonucleoprotein K | 1.61 |
| A_24_P944570 | PXDN | 1.61 |
| A_23_P124632 | IGHA1 | 1.6 |
| A_23_P145336 | HLA-DRB3 | 1.6 |
| A_23_P160940 | ABCA4 | 1.6 |
| A_23_P203751 | TMEM135 | 1.6 |
| A_23_P36448 | TMED2 | 1.6 |
| A_23_P77103 | SORD | 1.6 |
| A_24_P68649 | RNPEP | 1.6 |
| A_23_P10127 | SFRP1 | 1.59 |
| A_23_P21800 | Ig kappa locus | 1.59 |
| A_23_P43988 | DPYD | 1.59 |
| A_23_P86421 | NCOA4 | 1.59 |
| A_24_P233850 | SDHC | 1.59 |
| A_24_P272146 | IGKC | 1.59 |
| A_24_P357847 | Ig kappa locus | 1.59 |
| A_32_P159192 | ENST00000295339 | 1.59 |
| A_23_P10121 | SFRP1 | 1.58 |
| A_23_P158593 | COL5A1 | 1.58 |
| A_23_P211212 | COL18A1 | 1.58 |
| A_23_P216429 | ASPN | 1.58 |
| A_23_P428129 | CDKN1C | 1.58 |
| A_23_P45361 | GLUD2 | 1.58 |
| A_23_P98092 | OAT | 1.58 |
| A_24_P183664 | TRIL | 1.58 |
| A_24_P370472 | HLA-DRB4 | 1.58 |
| A_24_P472081 | ENST00000331195 | 1.58 |
| A_24_P750327 | Ig M heavy chain variable region | 1.58 |
| A_23_P108082 | CREB3L3 | 1.57 |
| A_23_P110175 | CTSO | 1.57 |
| A_23_P111000 | PSMB9 | 1.57 |
| A_23_P124300 | BCMO1 | 1.57 |
| A_23_P39465 | BST2 | 1.57 |
| A_24_P246591 | ENST00000305820 | 1.57 |
| A_24_P358321 | ENST00000377233 | 1.57 |
| A_24_P605563 | Anti-rabies SOJB immunoglobulin lambda light chain | 1.57 |
| A_23_P119562 | CFD | 1.56 |
| A_23_P156431 | MAN1A1 | 1.56 |
| A_23_P350782 | ENST00000307840 | 1.56 |
| A_23_P361654 | IGKC | 1.56 |
| A_23_P76322 | PIK3C2G | 1.56 |
| A_23_P84791 | A_23_P84791 | 1.56 |
| A_23_P90710 | DES | 1.56 |
| A_24_P260101 | MME | 1.56 |
| A_23_P120883 | HMOX1 | 1.55 |
| A_23_P211233 | COL6A2 | 1.55 |
| A_24_P190424 | RAB8A | 1.55 |
| A_24_P205589 | ACOT7 | 1.55 |
| A_32_P125832 | AK123079 | 1.55 |
| A_23_P112026 | INDO | 1.54 |
| A_23_P116235 | MDK | 1.54 |
| A_23_P133916 | C2 | 1.54 |
| A_23_P145841 | SOSTDC1 | 1.54 |
| A_23_P1759 | AMICA1 | 1.54 |
| A_23_P255111 | A_23_P255111 | 1.54 |
| A_23_P31844 | ATP6V1B2 | 1.54 |
| A_23_P40240 | CTSZ | 1.54 |
| A_23_P404698 | FLJ35880 | 1.54 |
| A_24_P284959 | PCDH18 | 1.54 |
| A_24_P490109 | A_24_P490109 | 1.54 |
| A_24_P83102 | IGLL1 | 1.54 |
| A_32_P148118 | ENST00000331696 | 1.54 |
| A_23_P155624 | AP2M1 | 1.53 |
| A_23_P169437 | LCN2 | 1.53 |
| A_23_P210100 | CYP26B1 | 1.53 |
| A_23_P213171 | MTTP | 1.53 |
| A_23_P217326 | FHL1 | 1.53 |
| A_23_P66854 | KRT20 | 1.53 |
| A_24_P174550 | RHOA | 1.53 |
| A_24_P243528 | HLA-DPA1 | 1.53 |
| A_24_P296772 | PPP1R14A | 1.53 |
| A_24_P307375 | ENST00000312946 | 1.53 |
| A_24_P33895 | ATF3 | 1.53 |
| A_24_P340036 | RNF128 | 1.53 |
| A_24_P401392 | A_24_P401392 | 1.53 |
| A_24_P409816 | A_24_P409816 | 1.53 |
| A_24_P76210 | THC2365247 | 1.53 |
| A_32_P100830 | A_32_P100830 | 1.53 |
| A_32_P234405 | PIGR | 1.53 |
| A_23_P159435 | BC107852 | 1.52 |
| A_23_P165848 | EMILIN1 | 1.52 |
| A_23_P21260 | ENST00000359488 | 1.52 |
| A_23_P37598 | NPTN | 1.52 |
| A_23_P67381 | SULT2A1 | 1.52 |
| A_24_P75708 | A_24_P75708 | 1.52 |
| A_24_P852601 | HNF4G | 1.52 |
| A_24_P881527 | CTNND1 | 1.52 |
| A_23_P110712 | DUSP1 | 1.51 |
| A_23_P158868 | A_23_P158868 | 1.51 |
| A_23_P159163 | A_23_P159163 | 1.51 |
| A_23_P202837 | CCND1 | 1.51 |
| A_23_P211039 | ADAMTS1 | 1.51 |
| A_23_P28238 | SNX17 | 1.51 |
| A_23_P401606 | EDIL3 | 1.51 |
| A_23_P56630 | STAT1 | 1.51 |
| A_23_P5778 | RAB17 | 1.51 |
| A_23_P61068 | A_23_P61068 | 1.51 |
| A_23_P80570 | AADAC | 1.51 |
| A_23_P86424 | NCOA4 | 1.51 |
| A_24_P15973 | A_24_P15973 | 1.51 |
| A_24_P222872 | UGT1A6 | 1.51 |
| A_24_P252934 | APOA4 | 1.51 |
| A_24_P337380 | HNRPH1 | 1.51 |
| A_24_P383660 | A_24_P383660 | 1.51 |
| A_24_P639701 | AY062331 | 1.51 |
| A_24_P673209 | A_24_P673209 | 1.51 |
| A_24_P813550 | ENST00000216649 | 1.51 |
| A_24_P846755 | A_24_P846755 | 1.51 |
| A_32_P107372 | GBP1 | 1.51 |
| A_32_P148710 | CFL1 | 1.51 |
| A_32_P162183 | C2 | 1.51 |
| A_32_P8666 | LOC644063 | 1.51 |
| A_24_P140171 | CRTAP | 1.5 |
| A_24_P239076 | CTA-246H3.1 | 1.5 |
| A_24_P246626 | ENST00000383097 | 1.5 |
| A_24_P305223 | CTAGE1 | 1.5 |
| A_24_P315854 | ENST00000216649 | 1.5 |
| A_24_P32646 | A_24_P32646 | 1.5 |
| A_24_P402242 | COL3A1 | 1.5 |

**Supplementary Table S1B. Gene probes downregulated in 47 ileal CD compared to 52 non-ileal CD (UC and control non-IBD) samples** (total number of probes = 195).

| **Agilent ID** | **Gene Name, Accession Number, Description** | **Fold-change** |
| --- | --- | --- |
| A_23_P362694 | C4orf7 | 0.23 |
| A_23_P121695 | CXCL13 | 0.26 |
| A_23_P357717 | TCL1A | 0.27 |
| A_23_P123853 | CCL19 | 0.34 |
| A_24_P940348 | FAM129C) also termed BCNP1 | 0.35 |
| A_24_P133905 | CCL23 | 0.36 |
| A_24_P621701 | THC2397697 | 0.37 |
| A_23_P124542 | CR2 | 0.39 |
| A_23_P46039 | FCRLM1 | 0.39 |
| A_23_P113572 | CD19 | 0.4 |
| A_23_P343398 | CCR7 | 0.4 |
| A_23_P10232 | BANK1 | 0.41 |
| A_24_P252945 | CXCR5 | 0.41 |
| A_23_P101407 | C3 | 0.42 |
| A_23_P116371 | MS4A1 | 0.42 |
| A_23_P253791 | CAMP | 0.45 |
| A_23_P115201 | FCRL4 | 0.47 |
| A_23_P102000 | CXCR4 | 0.48 |
| A_32_P216122 | AK130891 | 0.48 |
| A_32_P44394 | AIM2 | 0.49 |
| A_23_P160751 | FCRL2 | 0.5 |
| A_23_P31725 | BLK | 0.5 |
| A_23_P115200 | FCRL4 | 0.51 |
| A_23_P143935 | PIGZ | 0.51 |
| A_24_P254106 | FLJ22814 | 0.51 |
| A_23_P152926 | GP1BA | 0.53 |
| A_23_P26358 | SMG1 | 0.53 |
| A_23_P312920 | POU2AF1 | 0.53 |
| A_24_P932388 | DB340110 | 0.53 |
| A_32_P215143 | LOC647022 | 0.53 |
| A_32_P72067 | ARHGAP24 | 0.53 |
| A_23_P214208 | CNR1 | 0.54 |
| A_23_P358438 | FCRL3 | 0.54 |
| A_23_P500998 | HOXA9 | 0.54 |
| A_24_P328504 | SP140 | 0.54 |
| A_32_P34920 | FOXD1 | 0.54 |
| A_32_P48054 | ENST00000374472 | 0.54 |
| A_23_P115192 | FCRL4 | 0.55 |
| A_23_P166371 | VPREB3 | 0.55 |
| A_24_P941359 | C6orf32 | 0.55 |
| A_32_P209582 | LOC645238 | 0.55 |
| A_23_P149368 | FCRL1 | 0.56 |
| A_23_P201211 | FCRL5 | 0.56 |
| A_23_P7185 | BRDG1 | 0.56 |
| A_24_P741023 | BC008476 | 0.56 |
| A_23_P166848 | LTF | 0.57 |
| A_23_P363316 | HOXB5 | 0.57 |
| A_23_P435183 | ENST00000308482 | 0.57 |
| A_23_P70968 | HOXA7 | 0.57 |
| A_24_P276576 | FCRLM1 | 0.57 |
| A_24_P395415 | Translocation associated fusion protein IRTA1/IGA1 | 0.57 |
| A_24_P98948 | BU943730 | 0.57 |
| A_32_P111266 | THC2453189 | 0.57 |
| A_32_P164573 | THC2314215 | 0.57 |
| A_32_P43878 | THC2379275 | 0.57 |
| A_32_P85880 | A_32_P85880 | 0.57 |
| A_23_P139500 | BHLHB3 | 0.58 |
| A_23_P203558 | HBB | 0.58 |
| A_23_P371076 | KLF12 | 0.58 |
| A_23_P93772 | HOXA5 | 0.58 |
| A_32_P138409 | THC2442489 | 0.58 |
| A_23_P139500 | BHLHB3 | 0.58 |
| A_32_P8813 | LOC283663 | 0.58 |
| A_23_P29096 | PDE9A | 0.59 |
| A_24_P153576 | SHPRH | 0.59 |
| A_32_P211026 | THC2342793 | 0.59 |
| A_32_P37584 | AW340352 | 0.59 |
| A_23_P124934 | ZNFN1A1 | 0.6 |
| A_23_P339079 | ZNF573 | 0.6 |
| A_23_P383698 | LOC440345 | 0.6 |
| A_23_P4551 | SETBP1 | 0.6 |
| A_23_P49145 | ZG16 | 0.6 |
| A_23_P75915 | RIC3 | 0.6 |
| A_23_P99386 | TNFSF11 | 0.6 |
| A_24_P899020 | A_24_P899020 | 0.6 |
| A_32_P125589 | THC2343933 | 0.6 |
| A_32_P163594 | A_32_P163594 | 0.6 |
| A_32_P172545 | THC2315069 | 0.6 |
| A_32_P53976 | A_32_P53976 | 0.6 |
| A_32_P61298 | CDR1 | 0.6 |
| A_23_P156218 | GZMK | 0.61 |
| A_23_P16225 | VMD2L1 | 0.61 |
| A_23_P423074 | KIAA0888 | 0.61 |
| A_23_P7503 | TIMD4 | 0.61 |
| A_23_P98910 | LRMP | 0.61 |
| A_24_P140204 | PXK | 0.61 |
| A_24_P212457 | GON4L | 0.61 |
| A_24_P922261 | SRGAP1 | 0.61 |
| A_32_P162862 | LOC644891 | 0.61 |
| A_32_P190682 | THC2347318 | 0.61 |
| A_32_P208200 | THC2393794 | 0.61 |
| A_32_P225301 | THC2280343 | 0.61 |
| A_32_P45375 | AF037219 | 0.61 |
| A_32_P71171 | A_32_P71171 | 0.61 |
| A_23_P131024 | ZBTB32 | 0.62 |
| A_23_P156811 | LOC389286 | 0.62 |
| A_23_P206585 | PRKCB1 | 0.62 |
| A_23_P2901 | C14orf113 | 0.62 |
| A_23_P310931 | CNR2 | 0.62 |
| A_23_P366453 | KHDRBS2 | 0.62 |
| A_23_P372234 | CA12 | 0.62 |
| A_23_P39067 | SPIB | 0.62 |
| A_23_P391344 | RASGEF1A | 0.62 |
| A_24_P109432 | ENST00000339968 | 0.62 |
| A_24_P465879 | AK026466 | 0.62 |
| A_24_P50829 | TRPM7 | 0.62 |
| A_24_P595223 | C22orf35 | 0.62 |
| A_32_P137604 | BC018597 | 0.62 |
| A_32_P71876 | THC2438936 | 0.62 |
| A_32_P75399 | THC2308675 | 0.62 |
| A_23_P112470 | CCL21 | 0.63 |
| A_23_P115726 | SLC16A9 | 0.63 |
| A_23_P136753 | THC2275252 | 0.63 |
| A_23_P155257 | FOXP1 | 0.63 |
| A_23_P26325 | CCL17 | 0.63 |
| A_23_P26713 | RPL23 | 0.63 |
| A_23_P36641 | AICDA | 0.63 |
| A_23_P40174 | MMP9 | 0.63 |
| A_23_P418477 | AKAP11 | 0.63 |
| A_23_P66137 | SOX8 | 0.63 |
| A_24_P136807 | RFC1 | 0.63 |
| A_24_P187131 | FRYL | 0.63 |
| A_24_P37020 | THC2282321 | 0.63 |
| A_24_P529786 | AK091744 | 0.63 |
| A_32_P132766 | THC2406017 | 0.63 |
| A_32_P177725 | THC2416098 | 0.63 |
| A_32_P39963 | EXOSC6 | 0.63 |
| A_32_P6972 | THC2341837 | 0.63 |
| A_32_P88987 | AK022346 | 0.63 |
| A_23_P12082 | CHI3L2 | 0.64 |
| A_23_P351286 | FLJ22814 | 0.64 |
| A_23_P433785 | P2RX5 | 0.64 |
| A_24_P301954 | MGC16384 | 0.64 |
| A_24_P935682 | AY358248 | 0.64 |
| A_32_P133038 | THC2358845 | 0.64 |
| A_32_P147969 | THC2304550 | 0.64 |
| A_32_P169222 | THC2328913 | 0.64 |
| A_32_P30905 | WDFY4 | 0.64 |
| A_32_P33213 | THC2358845 | 0.64 |
| A_23_P15876 | ALPK2 | 0.65 |
| A_23_P159316 | BFSP2 | 0.65 |
| A_23_P201731 | TRAF5 | 0.65 |
| A_23_P207201 | CD79B | 0.65 |
| A_23_P258088 | PACSIN1 | 0.65 |
| A_23_P34018 | RPL39 | 0.65 |
| A_23_P44956 | LOC441136 | 0.65 |
| A_23_P500093 | RGS13 | 0.65 |
| A_24_P168925 | ENST00000372045 | 0.65 |
| A_24_P360529 | PDE7A | 0.65 |
| A_24_P409681 | A_24_P409681 | 0.65 |
| A_24_P883109 | TYW1 | 0.65 |
| A_24_P931859 | AK074662 | 0.65 |
| A_24_P937546 | CMTM7 | 0.65 |
| A_32_P110086 | A_32_P110086 | 0.65 |
| A_32_P117186 | CR749547 | 0.65 |
| A_32_P132337 | A_32_P132337 | 0.65 |
| A_32_P184727 | KPNB1 | 0.65 |
| A_32_P220580 | AK124352 | 0.65 |
| A_32_P221641 | A_32_P221641 | 0.65 |
| A_32_P42149 | A_32_P42149 | 0.65 |
| A_23_P127789 | AHNAK | 0.66 |
| A_23_P156809 | LOC389286 | 0.66 |
| A_23_P162668 | CPM | 0.66 |
| A_23_P210690 | TRIB3 | 0.66 |
| A_23_P215009 | C6orf32 | 0.66 |
| A_23_P218858 | ABI3BP | 0.66 |
| A_23_P30634 | BACH2 | 0.66 |
| A_23_P320216 | FAM55D | 0.66 |
| A_23_P321984 | DCAL1 | 0.66 |
| A_23_P405707 | BCOR | 0.66 |
| A_23_P52846 | THC2447689 | 0.66 |
| A_23_P79398 | IL1R2 | 0.66 |
| A_23_P85800 | CD52 | 0.66 |
| A_24_P136711 | BC030757 | 0.66 |
| A_24_P20630 | LEF1 | 0.66 |
| A_24_P214598 | PPM1K | 0.66 |
| A_24_P830025 | FNTA | 0.66 |
| A_24_P910490 | THC2340757 | 0.66 |
| A_24_P927222 | ENST00000265271 | 0.66 |
| A_24_P940079 | A_24_P940079 | 0.66 |
| A_32_P118556 | A_32_P118556 | 0.66 |
| A_32_P151244 | AK022268 | 0.66 |
| A_32_P160615 | A_32_P160615 | 0.66 |
| A_32_P174285 | THC2442021 | 0.66 |
| A_32_P220161 | BI497361 | 0.66 |
| A_32_P51707 | THC2348290 | 0.66 |
| A_32_P57057 | USP15 | 0.66 |
| A_32_P80016 | N47124 | 0.66 |
| A_23_P70069 | AK000420 | 0.67 |
| A_24_P107918 | CBFB | 0.67 |
| A_24_P155761 | AKAP13 | 0.67 |
| A_24_P75190 | HBD | 0.67 |
| A_24_P829934 | THC2341944 | 0.67 |
| A_24_P926025 | DKFZp547E087 | 0.67 |
| A_32_P86494 | A_32_P86494 | 0.67 |
