# Supplementary material for: An Ileal Crohn's Disease Gene Signature Based on Whole Human Genome Expression Profiles of Disease Unaffected Ileal Mucosal Biopsies
Source: PLoS One. 2012 May 14;7(5):e37139. doi: 10.1371/journal.pone.0037139 (PMC3351422; doi:10.1371/journal.pone.0037139)
Supplement: Table S2 — Union of differentially expressed Agilent gene probes selected by four feature subset selection methods: Boosting, PAM, Random Forest (RF) and LASSO. A. Upregulated probes B. Downregulated probes The 17 genes selected by the boosting method are bolded. (DOCX) [file pone.0037139.s002.docx]

**Supplementary Table S2A.** **Union of the upregulated Agilent gene probes selected by four feature subset selection methods: Boosting, PAM, Random Forest (RF) and LASSO**. The 17 genes selected by the boosting method are **bolded.**

| **Agilent Probe ID** | **Gene Accession ID** | **Gene Description** | **Boosting** | **PAM** | **RF** | **LASSO** | **Training Set Fold Change** | **Test Set Fold Change** |
| --- | --- | --- | --- | --- | --- | --- | --- | --- |
| **A_23_P47616** | **NM_004476** | **Folate hydrolase 1 (*FOLH1*)** | **X** | **X** | **X** | **X** | **6.26** | **2.10** |
| **A_24_P183664** | **NM_014817** | **TLR4 interactor with leucine rich repeats (*TRIL*)** | **X** |  | **X** | **X** | **1.58** | **1.88** |
| A_23_P20075 | NM_013389 | Niemann-Pick disease, type C1, gene-like 1 (*NPC1L1*) |  | X | X | X | 3.1 | 2.72 |
| **A_23_P208706** | **NM_138764** | **BCL2-associated X protein (*BAX*)** | **X** |  |  | **X** | **1.7** | **1.91** |
| **A_23_P210100** | **NM_019885** | **Cytochrome P 450, family 26, subfamily B, polypeptide 1 (*CYP26B1*)** | **X** |  |  | **X** | **1.53** | **1.02** |
| **A_23_P253896** | **NM_001033047** | **Nephronectin (*NPNT*)** | **X** |  |  | **X** | **1.72** | **1.37** |
| **A_24_P296772** | **NM_033256** | **Protein phosphatase 1, regulatory (inhibitor) subunit 14A (*PPP1R14A*)** | **X** |  |  | X | 1.53 | 1.70 |
| A_32_P157391 | NM_153696 | Folate hydrolyase 1B (*FOLH1B*) |  | X | X |  | 5.35 | 2.46 |
| A_23_P106194 | NM_005252 | FBJ murine osteosarcoma viral oncogene homolog (*FOS*) |  | X |  | X | 2.28 | 0.97 |
| **A_23_P77103** | **NM_003104** | **Sorbitol dehydrogenase (*SORD*)** | **X** |  |  |  | **1.6** | **1.92** |
| **A_23_P145841** | **NM_015464** | **Sclerostin domain containing 1 (*SOSTDC1*)** | **X** |  |  |  | **1.54** | **1.30** |
| **A_24_P698136** | **AK125299** | **Cytosolic acyl coenzyme A thioester hydrolase, similar (*LOC344967*)** | **X** |  |  |  | **1.63** | **1.29** |
| **A_24_P273679** | **NM_006106** | **Yes associated protein 1 (*YAP1*)** | **X** |  |  |  | **1.68** | **1.06** |
| **A_32_P100830** | **A_32_P100830** | **Unknown** | **X** |  |  |  | **1.53** | **1.79** |
| A_23_P79217 | NM_002299 | Lactase (*LCT*) |  | X |  |  | 3.69 | 2.91 |
| A_23_P10121 | NM_003012 | Secreted frizzled-related protein 1 (*SFRP1*) |  |  |  | X | 1.58 | 2.78 |
| A_23_P162739 | NM_183422 | TSC22 domain family, member 1 (*TSC22D1*) |  |  |  | X | 1.68 | 0.87 |

**Supplementary Table S2B.** **Union of the downregulated Agilent gene probes selected by the four feature subset selection methods: Boosting, PAM, Random Forest (RF) and LASSO**.

| **Agilent Probe ID** | **Gene Accession** | **Gene Description** | **Boosting** | **PAM** | **RF** | **LASSO** | **Training Set Fold Change** | **Test Set Fold Change** |
| --- | --- | --- | --- | --- | --- | --- | --- | --- |
| **A_32_P216122** | **AK130891** | **Unknown** | **X** | **X** | **X** |  | **0.48** | **0.76** |
| A_23_P362694 | NM_152997 | *C4orf7* also termed follicular dendritic cell secreted protein |  | X | X | X | 0.23 | 0.32 |
| A_24_P932388 | DB340110 | Unknown |  | X | X | X | 0.53 | 0.84 |
| **A_23_P363316** | **NM_002147** | **Homeobox B5 (*HOXB5*)** | **X** |  | **X** |  | **0.57** | **0.64** |
| **A_24_P883109** | **AL833452** | **tRNA-yW synthesizing protein 1 homolog (*TYW1*)** | **X** |  |  | X | 0.65 | 0.91 |
| A_24_P940348 | NM_173544 | Family with sequence similarity129, member C (*FAM129C*) also termed B-cell novel protein 1 (*BCNP1*) |  | X | X |  | 0.35 | 0.47 |
| A_23_P253791 | NM_004345 | Cathelicidin antimicrobial peptide (*CAMP*) |  | X | X |  | 0.45 | 0.55 |
| A_24_P133905 | NM_005064 | Chemokine (C-C motif) ligand 23 (*CCL23)* |  | X |  | X | 0.36 | 0.63 |
| A_23_P2901 | AK000041 | Unknown |  |  | X | X | 0.62 | 0.61 |
| **A_23_P258088** | **NM_020804** | **Protein kinase C and casein kinase substrate in neurons 1 (*PACSIN1*)** | **X** |  |  |  | **0.65** | **0.78** |
| **A_32_P172545** | **THC2315069** | **Unknown** | **X** |  |  |  | **0.60** | **0.93** |
| **A_32_P61298** | **AK054921** | **40S ribosomal protein S15A, similar (*CDR1*)** | **X** |  |  |  | **0.60** | **0.95** |
| A_24_P252945 | NM_032966 | Chemokine (C-X-C motif) receptor 5 (*CXCR5*) |  | X |  |  | 0.41 | 0.53 |
| A_23_P113572 | NM_001770 | CD19 molecule (*CD19*) |  | X |  |  | 0.4 | 0.46 |
| A_23_P121695 | NM_006419 | Chemokine (C-X-C motif) ligand 13 (*CXCL13*) |  | X |  |  | 0.26 | 0.40 |
| A_23_P46039 | NM_032738 | Fc receptor-like A (*FCRLA*) |  | X |  |  | 0.39 | 0.47 |
| A_23_P357717 | NM_021966 | T-cell leukemia/lymphoma 1A (*TCL1A*) |  | X |  |  | 0.27 | 0.38 |
| A_24_P621701 | THC2397697 | Unknown |  | X |  |  | 0.37 | 0.44 |
| A_32_P39963 | CR604283 | Exosome component 6 (*EXOSC6*) |  |  | X |  | 0.63 | 1.11 |
| A_23_P155257 | NM_032682 | Forkhead box P1 (*FOXP1*) |  |  | X |  | 0.63 | 0.85 |
| A_32_P209582 | XM_930310 | Unknown |  |  | X |  | 0.55 | 0.67 |
| A_24_P922261 | BC029919 | SLIT-ROBO Rho GTPase activating protein 1 (*SRGAP1*) |  |  | X |  | 0.61 | 0.94 |
| A_32_P164573 | THC2314215 | Unknown |  |  | X |  | 0.57 | 0.75 |
| A_32_P169222 | THC2328913 | Unknown |  |  | X |  | 0.64 | 0.82 |
| A_23_P101407 | NM_000064 | Complement component 3 (*C3*) |  |  |  | X | 0.41 | 0.50 |
