# Supplementary material for: An Ileal Crohn's Disease Gene Signature Based on Whole Human Genome Expression Profiles of Disease Unaffected Ileal Mucosal Biopsies
Source: PLoS One. 2012 May 14;7(5):e37139. doi: 10.1371/journal.pone.0037139 (PMC3351422; doi:10.1371/journal.pone.0037139)
Supplement: Table S3 — AUC values of empirical ROC curves, AUC values and 95% confidence interval (C.I.) of smoothed ROC curves. (DOCX) [file pone.0037139.s003.docx]

Supplementary Table S3. AUC values of empirical ROC curves, AUC values and 95% confidence interval (C.I.) of smoothed ROC curves.

| **Classifier** | **AUC of empirical ROC** | **AUC of smoothed ROC** | **(95%C.I. of smoothed ROC)** |
| --- | --- | --- | --- |
| **Training set** |  |  |  |
| Support Vector Machine (SVM) | 0.935 | 0.923 | (0.884, 0.986) |
| Random Forest (RF) | 0.915 | 0.899 | (0.854, 0.976) |
| Linear Discriminant Analysis (LDA) | 0.940 | 0.931 | (0.892, 0.988) |
| Predictive Analysis of Microarray (PAM) | 0.917 | 0.904 | (0.859, 0.975) |
| Lasso | 0.947 | 0.938 | (0.902, 0.992) |
| Boosting | 0.929 | 0.918 | (0.876, 0.982) |
| Naïve Bayes | 0.918 | 0.906 | (0.861, 0.976) |
| Combined Classifiers | 0.928 | 0.913 | (0.872, 0.983) |
|  |  |  |  |
| **Testing Set** |  |  |  |
| Support Vector Machine (SVM) | 0.905 | 0.876 | (0.796, 1) |
| Random Forest (RF) | 0.955 | 0.888 | (0.864, 1) |
| Linear Discriminant Analysis (LDA) | 0.910 | 0.895 | (0.870, 1) |
| Predictive Analysis of Microarray (PAM) | 0.980 | 0.952 | (0.942, 1) |
| Lasso | 0.905 | 0.897 | (0.760, 1) |
| Boosting | 0.955 | 0.944 | (0.884 ,1) |
| Naive Bayes | 0.980 | 0.952 | (0.942, 1) |
| Combined Classifiers | 0.955 | 0.942 | (0.880, 1) |
